# Supplementary material for: Structural insight into the activation mechanism of MrgD with heterotrimeric Gi-protein revealed by cryo-EM
Source: Commun Biol. 2022 Jul 15;5:707. doi: 10.1038/s42003-022-03668-3 (PMC9287403; doi:10.1038/s42003-022-03668-3)
Supplement: Supplementary file 2 — Supplementary Information [file 42003_2022_3668_MOESM2_ESM.pdf]

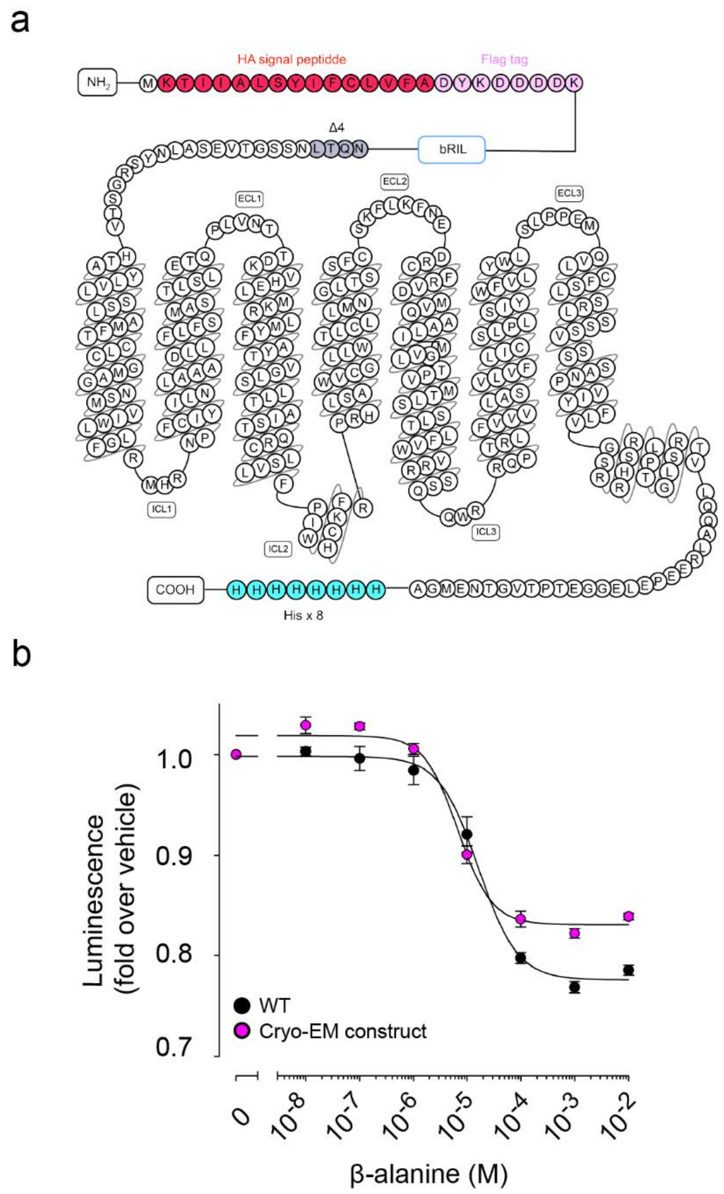

Supplementary Fig. 1 | Construct of the MrgD

a Snake plot of the MrgD construct used in this paper. **b** Comparison of β-alanine induced Gi signaling potency between WT and cryo-EM construct. The Gi coupling activaties were measured by the NanoBiT-G-protein dissociation assay. Dose-response curves are shown as means ± s.e.m. (standard error of the mean) of six independent experiments for WT and three for the cryo-EM construct

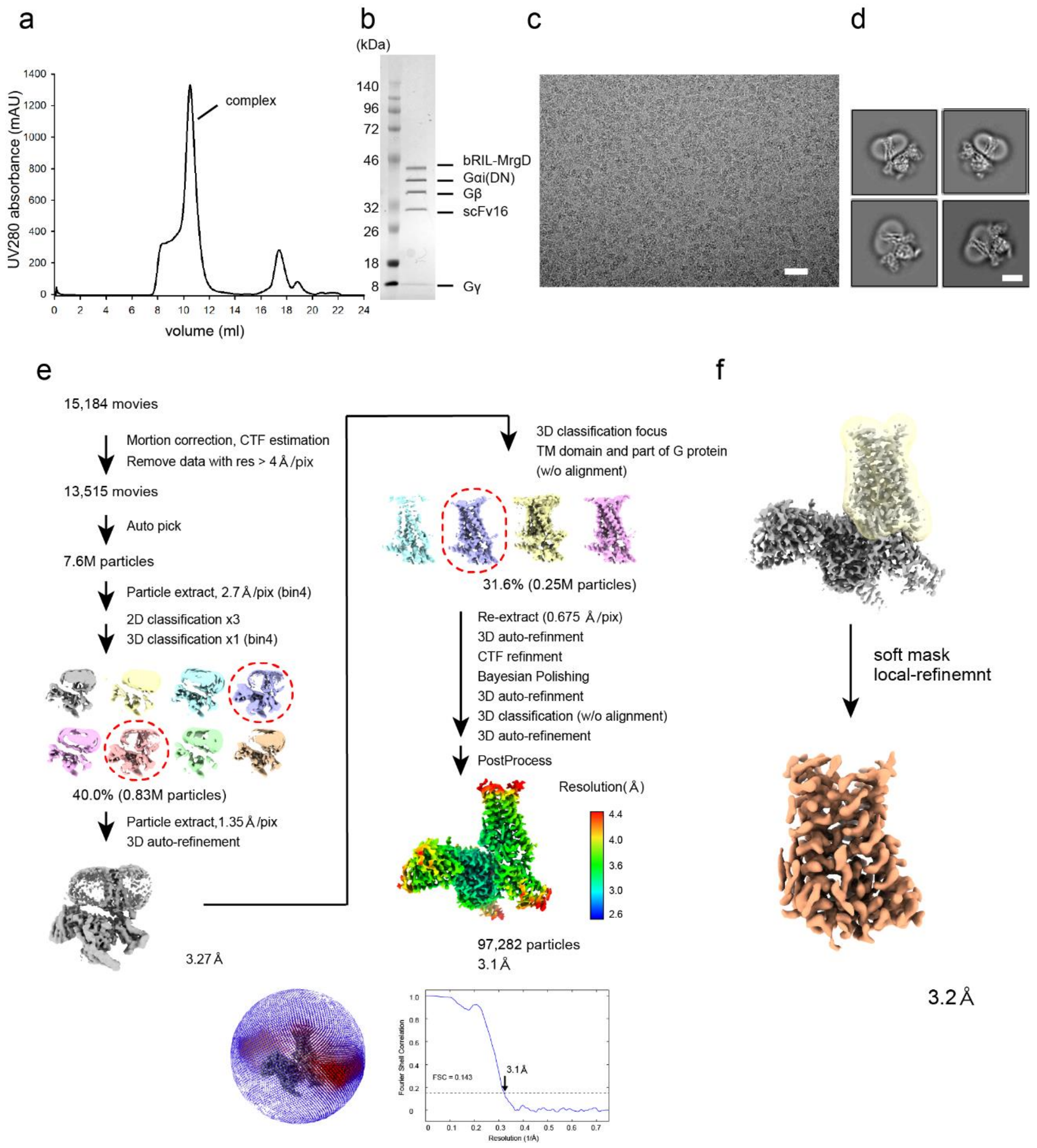

Supplementary Fig. 2 | Purification and flow chart of cryo-EM single-particle reconstruction of β-alanine-bound -MrgD-Gi complex.

a Elution profile of size-exclusion chromatography of the purified β-alanine-bound MrgD-Gi complex. **b** SDS–PAGE and Coomassie brilliant blue stain of the purified complex. **c** A representative micrograph of the β-alanine-bound MrgD-Gi complex (scale bar 30 nm). **d** Representative 2D class average images of the β-alanine-bound MrgD-Gi complex (scale bar 5 nm). **e** Flow chart of the single-particle analysis of the apo-MrgD-Gi complex. Fourier shell correlation (FSC) curve shows 3.1 Å global resolution according to the gold standard criteria

(FSC=0.143). Colors indicate local resolution on the cryo-EM map. **f** Local refinement was performed using a cryoSPARC. The density of the TM domain was significantly improved.

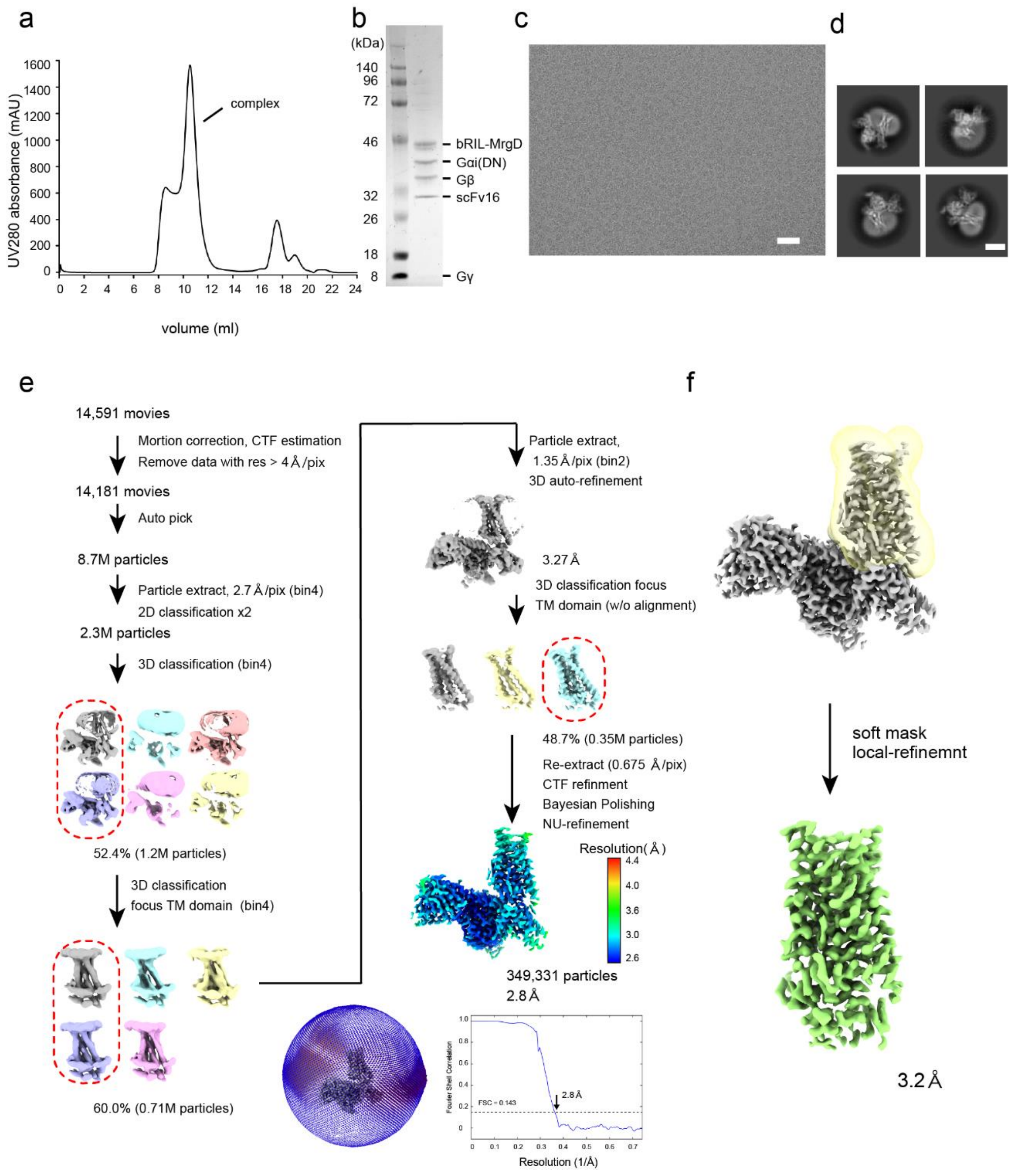

Supplementary Fig. 3 | Purification and flow chart of cryo-EM single-particle reconstruction of apo MrgD-Gi complex.

a Elution profile of size-exclusion chromatography of the purified apo MrgD-Gi complex. **b** SDS–PAGE and Coomassie brilliant blue stain of the purified complex. **c** A representative micrograph of the apo MrgD-Gi complex (scale bar 30 nm). **d** Representative 2D class average images of the apo MrgD-Gi complex (scale bar 5 nm). **e** Flow chart of the single-particle analysis of the apo MrgD-Gi complex. Fourier shell correlation (FSC) curve shows 2.8 Å global resolution according

to the gold standard criteria (FSC=0.143). Colors indicate local resolution on the cryo-EM map. **f** Local refinement was performed using a cryoSPARC. The density of the TM domain was significantly improved.

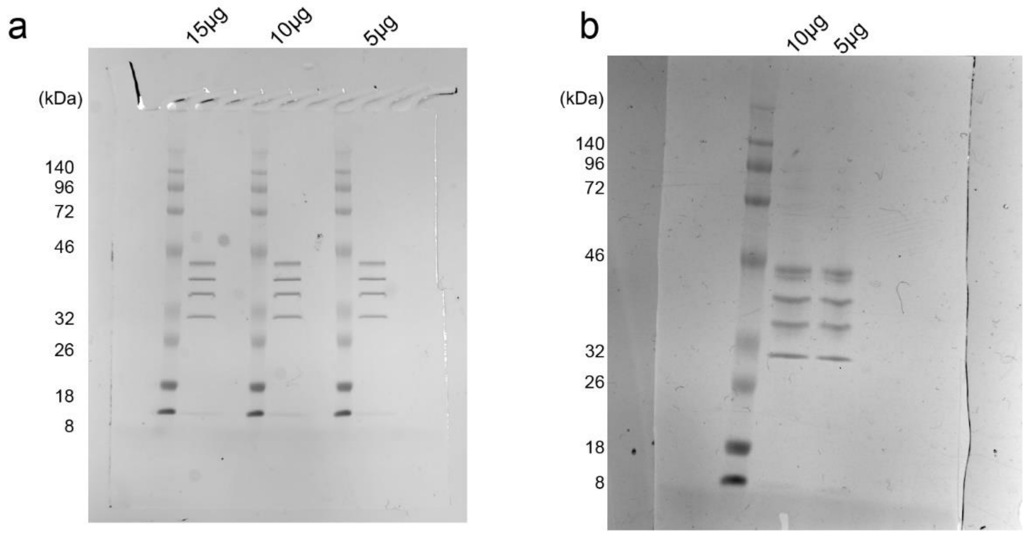

Supplementary Fig. 4 | uncropped SDS-PAGE gel related supplementary Fig.2b and 3b

SDS-PAGE of the purified MrgD-Gi complex. β-alanine bound state (a), apo state (b)

a

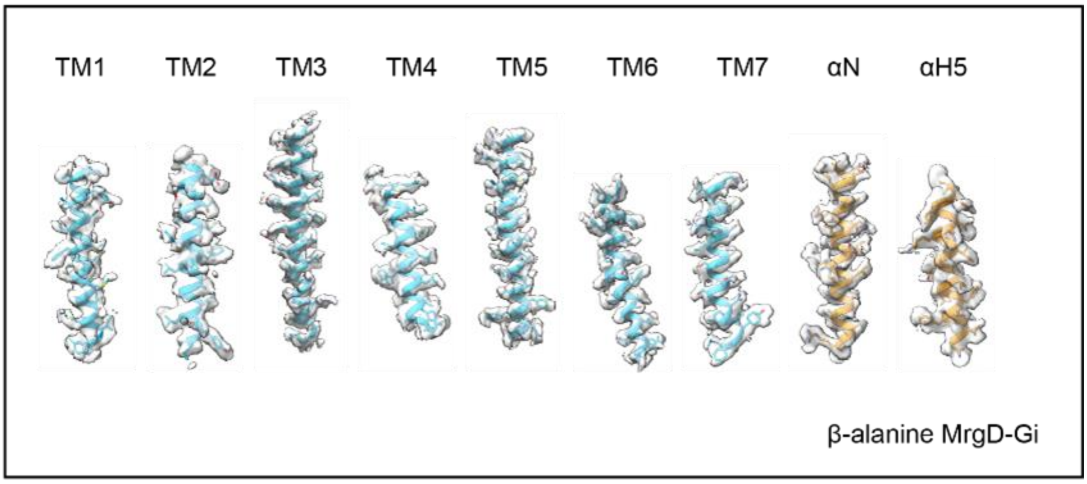

b

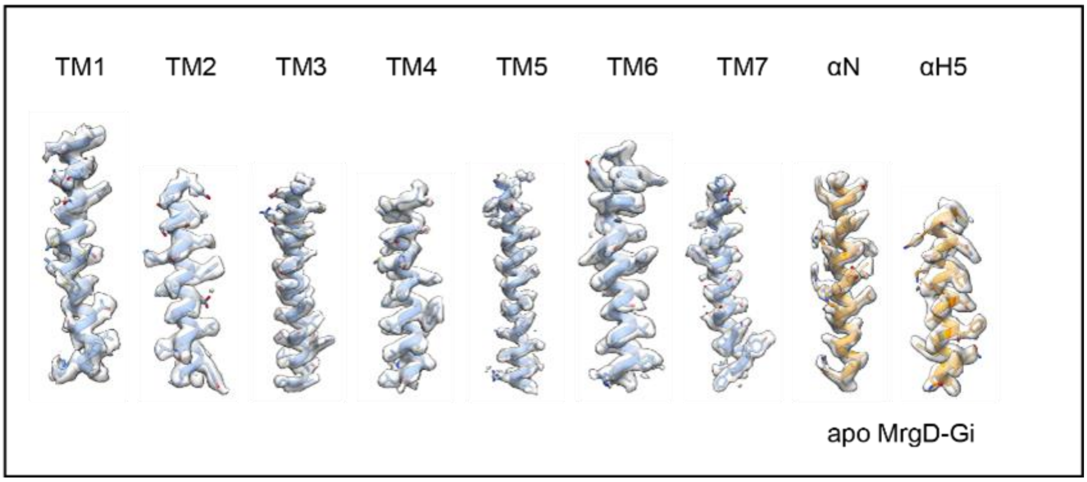

Supplementary Fig. 5 | Cryo-EM density maps of the β-alanine MrgD-Gi complex and apo MrgD-Gi complex.

a,b Cryo-EM density maps of the seven transmembrane helices and αN and α5 helices of Gαi. The β-alanine-bound MrgD-Gi complex is shown in (a), and the apo MrgD-Gi complex in (b).

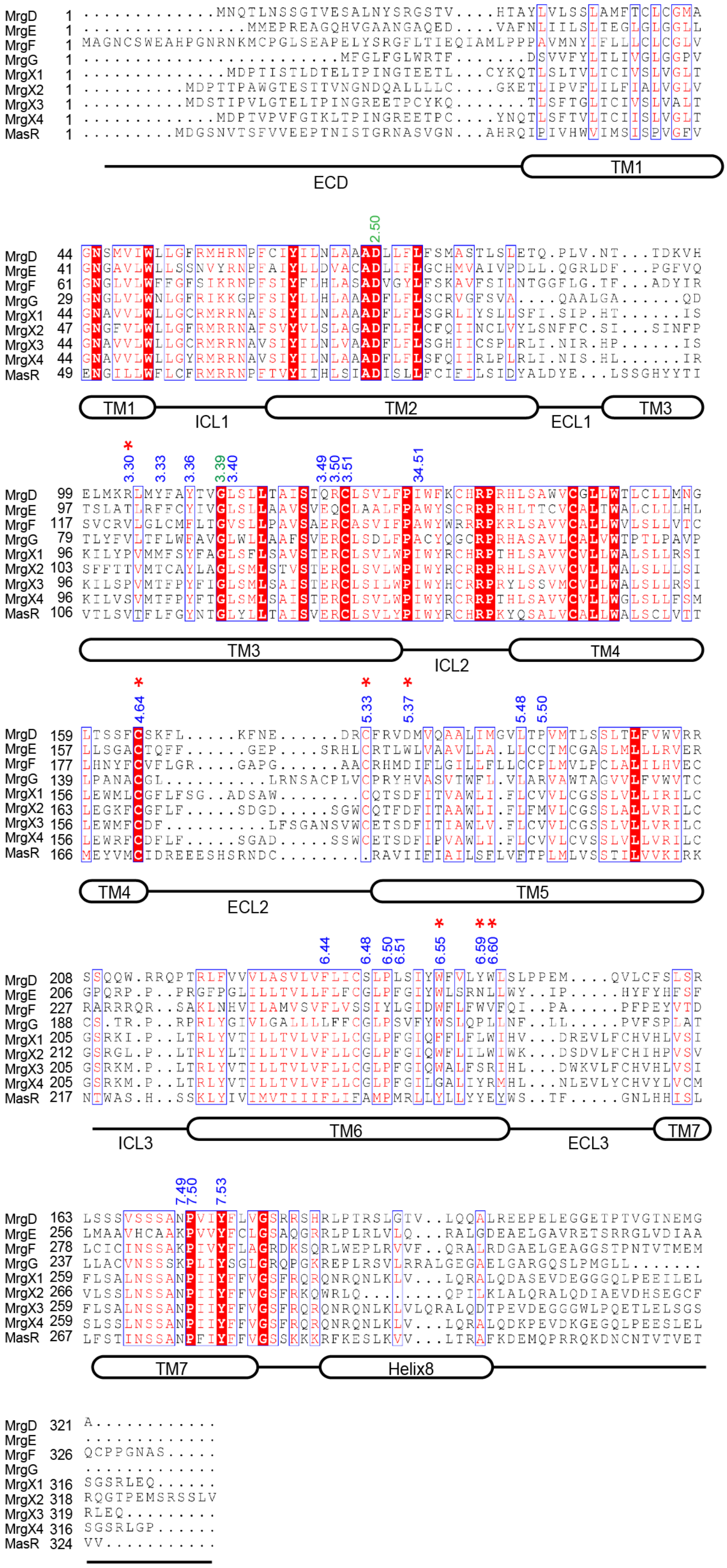
MrgD
MrgE
MrgF
MrgG
MrgX1
MrgX2
MrgX3
MrgX4
MasR
ECD
TM1
ICL1
TM2
ECL1
TM3
ICL2
TM4
ECL2
TM5
ICL3
TM6
ECL3
TM7
Helix8
2.50
3.30
3.33
3.36
3.39
3.40
3.49
3.50
3.51
34.51
4.64
5.33
5.37
5.48
5.50
6.44
6.48
6.50
6.51
6.55
6.59
6.60
7.49
7.50
7.53

Supplementary Fig. 6 | Sequence alignment of MrgD family proteins

The amino acid sequences of the MRGPR family proteins were aligned with GPCRdb (http://www.gpcrdb. org). The residues of the β-alanine binding sites are highlighted with red asterisks. Canonical sodium binding sites are highlighted with green. Superscripts refer to the Ballesteros-Weinstein numbers. The ovals represent the helical secondary structures based on MrgD.

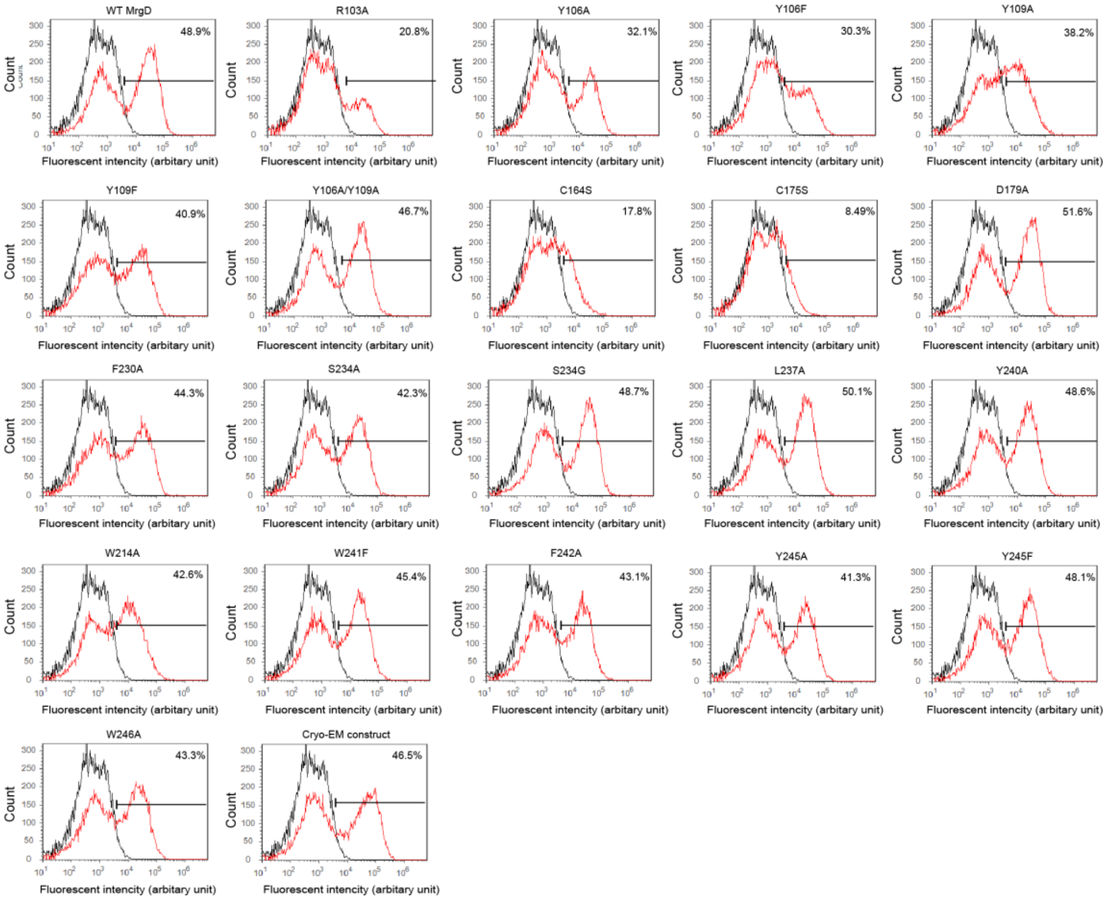

Supplementary Fig. 7 | Cell-surface expression of WT and mutant MrgD receptors

Cell-surface expression of MrgD analyzed by flow cytometry. Mock transfection and MrgD receptors are shown as black and red histograms, respectively. The black marker designates FLAG positive events. The gated percentage is described in the upper right. The expression levels were decreased by the R103A, C164S, and C175S.

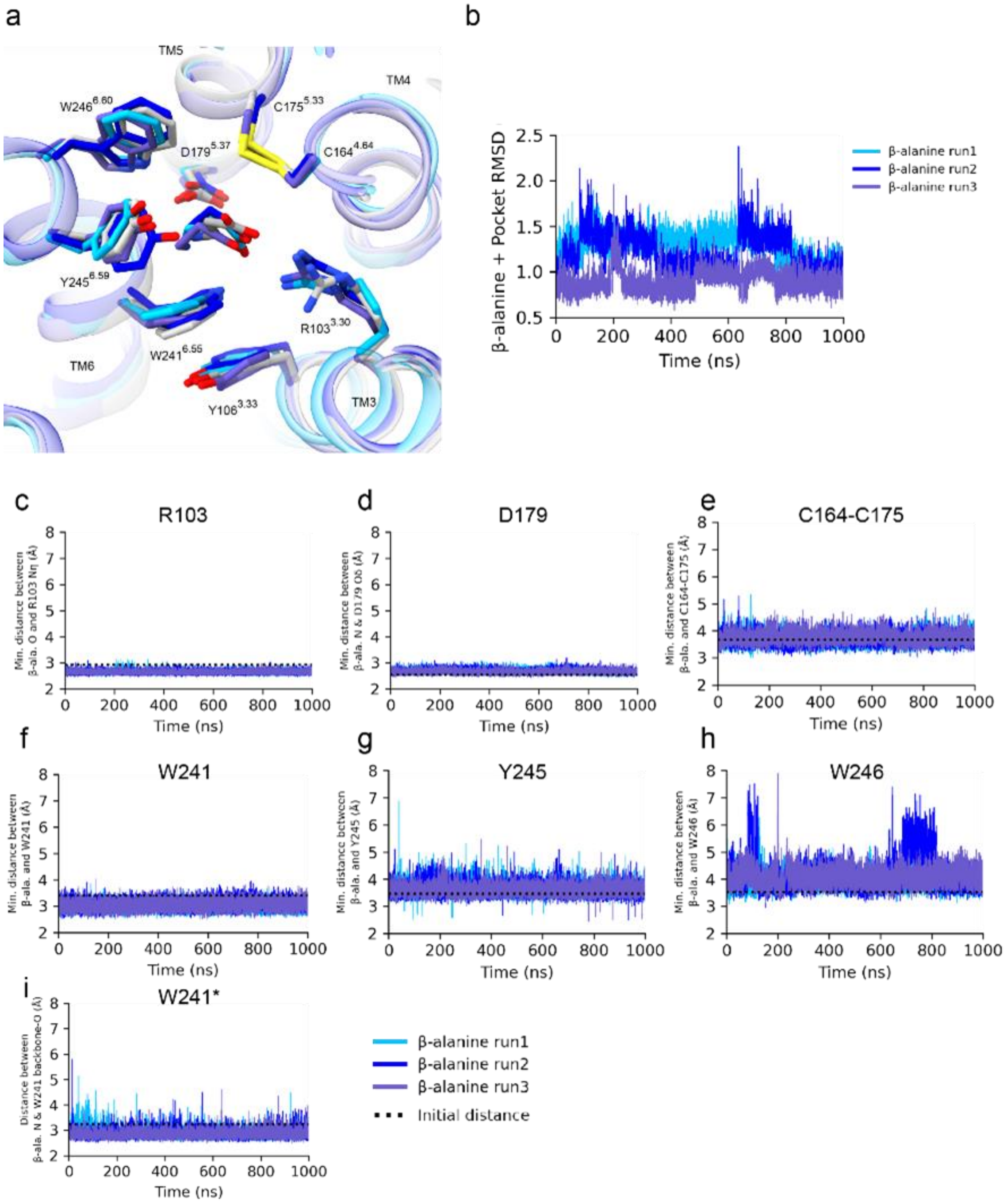

Supplementary Fig. 8 | MD simulations to assess the binding mode of β-alanine

a Superposition of the cryo-EM structure (grey) and the last snapshots from the three independent 1 μs MD simulations (run1: cyan, run2: blue, run3: slate blue). The snapshots show only minor changes in the ligand-binding pocket compared to the cryo-EM structure. **b** RMSD time course of the atom coordinates of the β-alanine and the residues comprising the binding pocket (R103, C164, C175, D179, W241, Y245 and W246), except hydrogens. The cryo-EM model was used as the

reference for the RMSD calculation. **c-i** Plots of the distances between the β-alanine and the surrounding residues. The initial distances measured in the cryo-EM model were shown as dashed lines. **c** Minimum distance between the two carboxyl O atoms of the β-alanine and the two Nη atoms in R103. **d** Minimum distance between the amino N atom of the β-alanine and the two Oδ atoms of D179. **e-h** Minimum distance between all atoms except hydrogens of the β-alanine and the side chain atoms except Cβ and hydrogens. **i** Distance between the amino N atom of the β-alanine and the backbone O atom of W241.

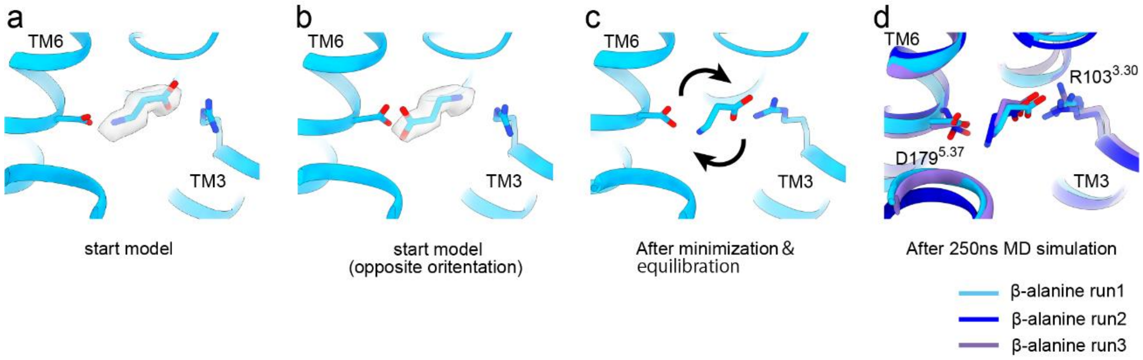

Supplementary Fig. 9 | MD simulation of β-alanine-bound MrgD started from the oppositely orientated β-alanine

a Original model of β-alanine-bound MrgD. **b** The model of β-alanine-bound MrgD with the β-alanine orientation intentionally reversed. **c** The structure after the energy-minimization and the equilibration. The β-alanine orientation spontaneously reverted back to the original orientation during the equilibration in all three independent runs. Only one of the three runs is shown in the figure for clarity. **d** The structures after the 250 ns production MD run. The orientations of the β-alanine remained the same as in the original model.

.

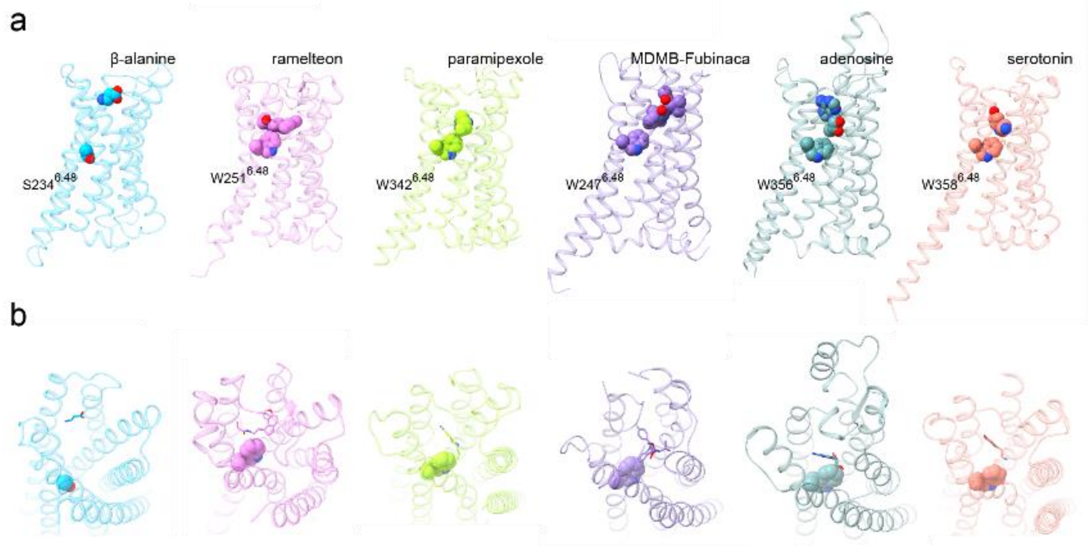

Supplementary Fig. 10 | Ligand binding positions of GPCRs

Agonists bound to MrgD and other class A GPCRs are shown as cartoon models. β-alanine (cyan, MrgD), ramelteon (pink, MT1R, PDB code: 7DB6), pramipexole (light green, D3R, PDB code: 7CMU), MDMB-Fubinaca (purple, CB1R, PDB code: 6N4B), adenosine (cadet blue, A1R, PDB code: 7LD4), serotonin (light orange, $5HT_{1A}$ PDB code: 7E2Y), and the residues at position 6.48 are shown as sphere models. (a) side view, (b) top view

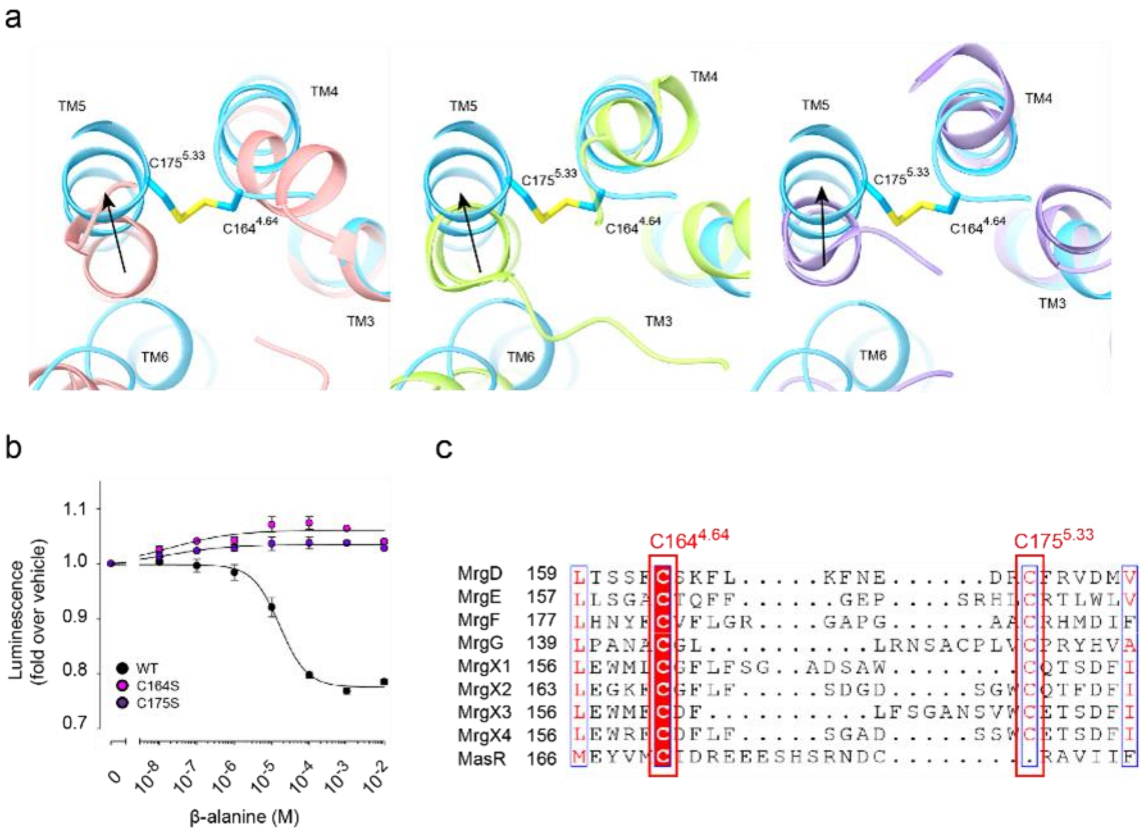

Supplementary Fig. 11 | A disulfide bond is a component of the ligand-binding pocket

a Comparison of TM4-TM5 helix arrangement with other class A GPCRs. MrgD was compared to AT1R (salmon, PDB code: 6OS0) (left), D3R (green, PDB code: 7CNU) (middle), and CB1 (purple, PDB code: 6N4B) (right) **b** NanoBiT-G-protein dissociation assays for concentration-response curves of G-protein dissociation signals are shown for WT MrgD (black) and C164S (magenta), and C174S (purple). Symbols and error bars represent the mean ± s.e.m of six independent experiments for WT and three for the mutants. **c** Sequence alignment of the MRGPR family showing cysteines involved in a disulfide bond. $C^{4.64}$ and $C^{5.33}$ are conserved in all MRGPR family proteins. Data for the graphs in **d** are available as Supplementary Data 1

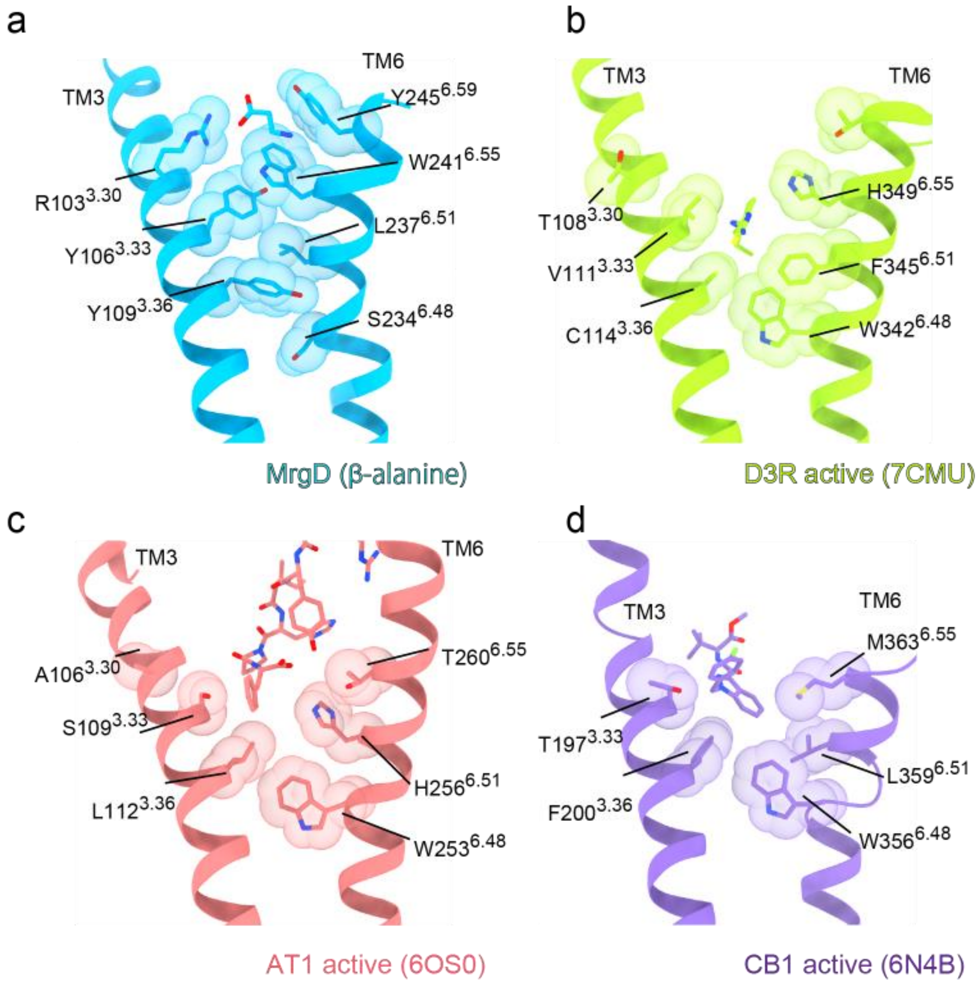

Supplementary Fig. 12 | Ligand binding site and activation switch

a-d Side views of ligand binding site and activation switch of MrgD with β-alanine (a), D3R (green, PDB code: 7CNU) with pramipexole (b), AT1R (salmon, PDB code: 6OS0) with Angiotensin II (c), CB1 (purple, PDB code: 6N4B) with MDMB-Fubinaca (d). The ligands and interacting residues are shown as stick models. Van der Waals surfaces are shown for the interacting residues.

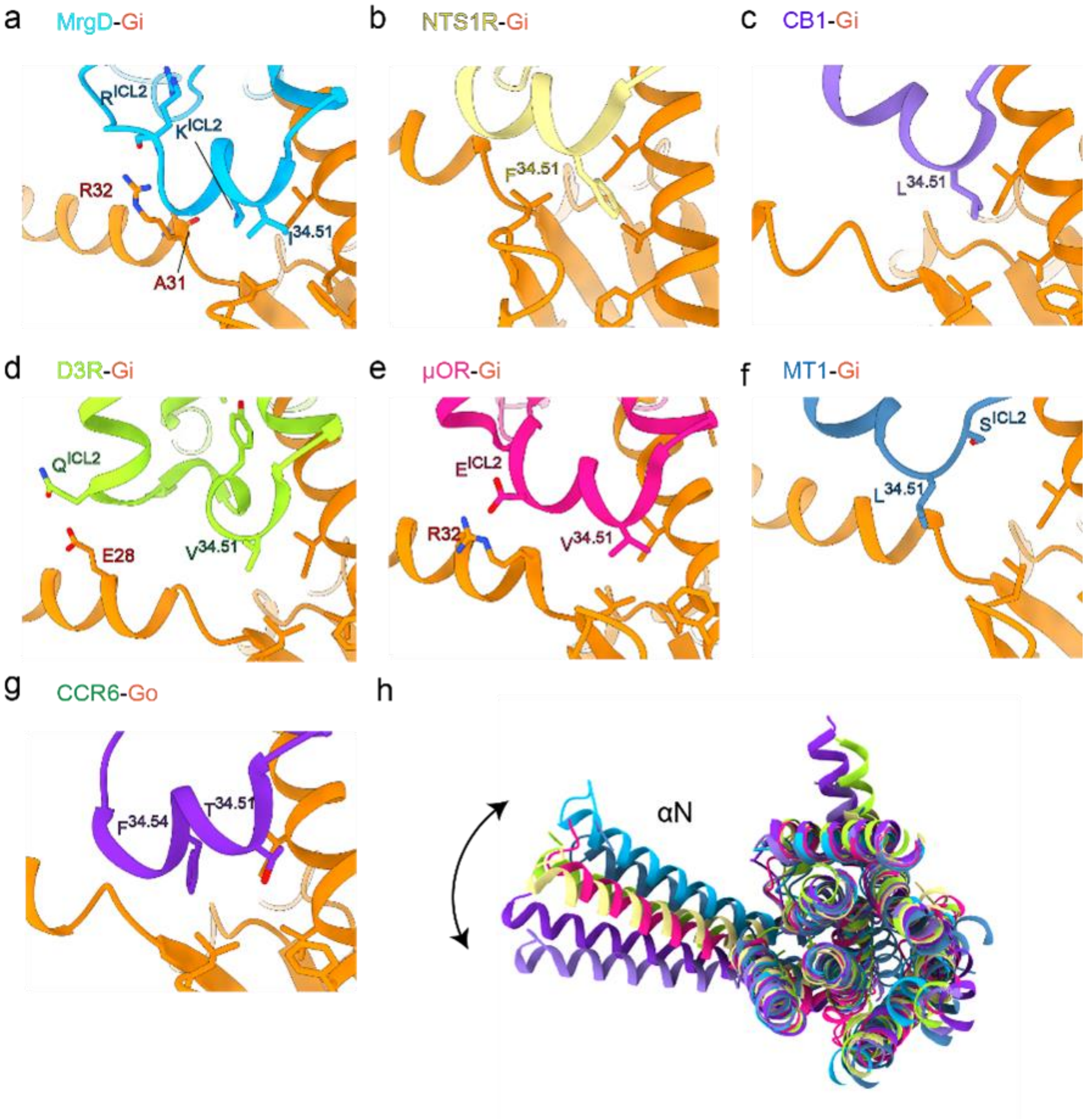

Supplementary Fig. 13 | Structural comparison of different GPCR-Gi protein interface

a-g Interactions between ICL2 of Receptor and aN-a5 of Gi. MrgD-Gi (cyan) (a), NTS1R–Gi (yellow, PDB code; 6OS9) (b), CB1-Gi (orchid, PDB code; 6N4B) (c), D3R-Gi (green, PDB code; 7CMU) (d), μOR-Gi (pink, PDB code; 6DDE) (e), MT1-Gi (blue, PDB code; 7DB6) (f), and CCR6-Go (purple, PDB code; 6WWZ) (g) are represented. **h** Difference in the angle of αN to Gi. Colors are consistent with (a) to (g).

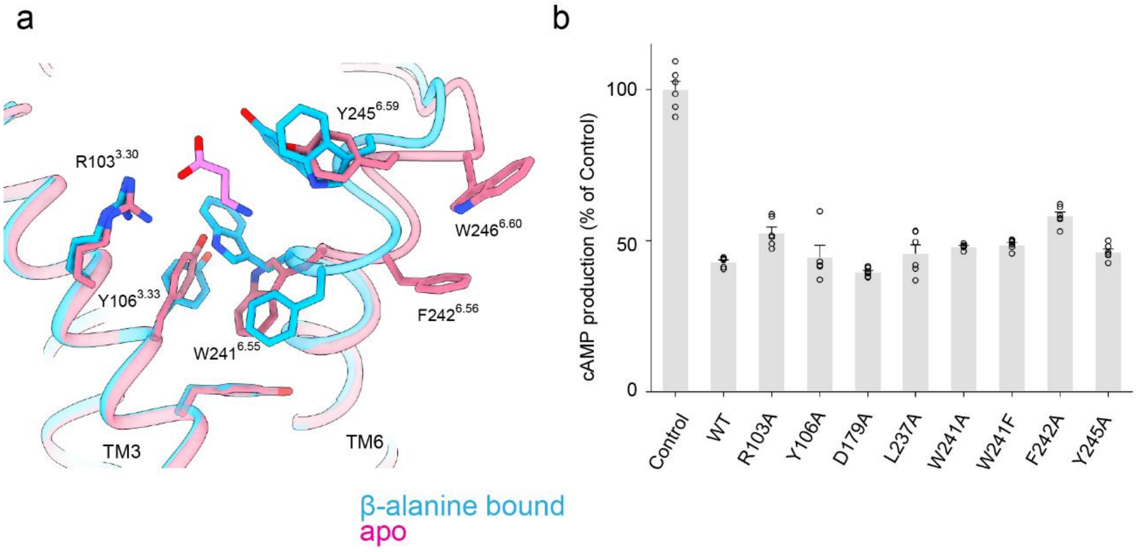

Supplementary Fig. 14 | Basal activity of MrgD with mutations around the ligand-binding site

a Magnified view of the ligand-binding pocket of β-alanine-bound MrgD (cyan) and the apo MrgD (red). The residues of interest are shown as sticks. **b** Basal activity of WT MrgD and mutants measured by cAMP inhibition assay. Bars represent mean ± s.e.m (n=5-6). The expression levels of these mutants at the cell surface were comparable to the wild type (Supplementary Fig.6). Data for the graphs in **d** are available as Supplementary Data 2

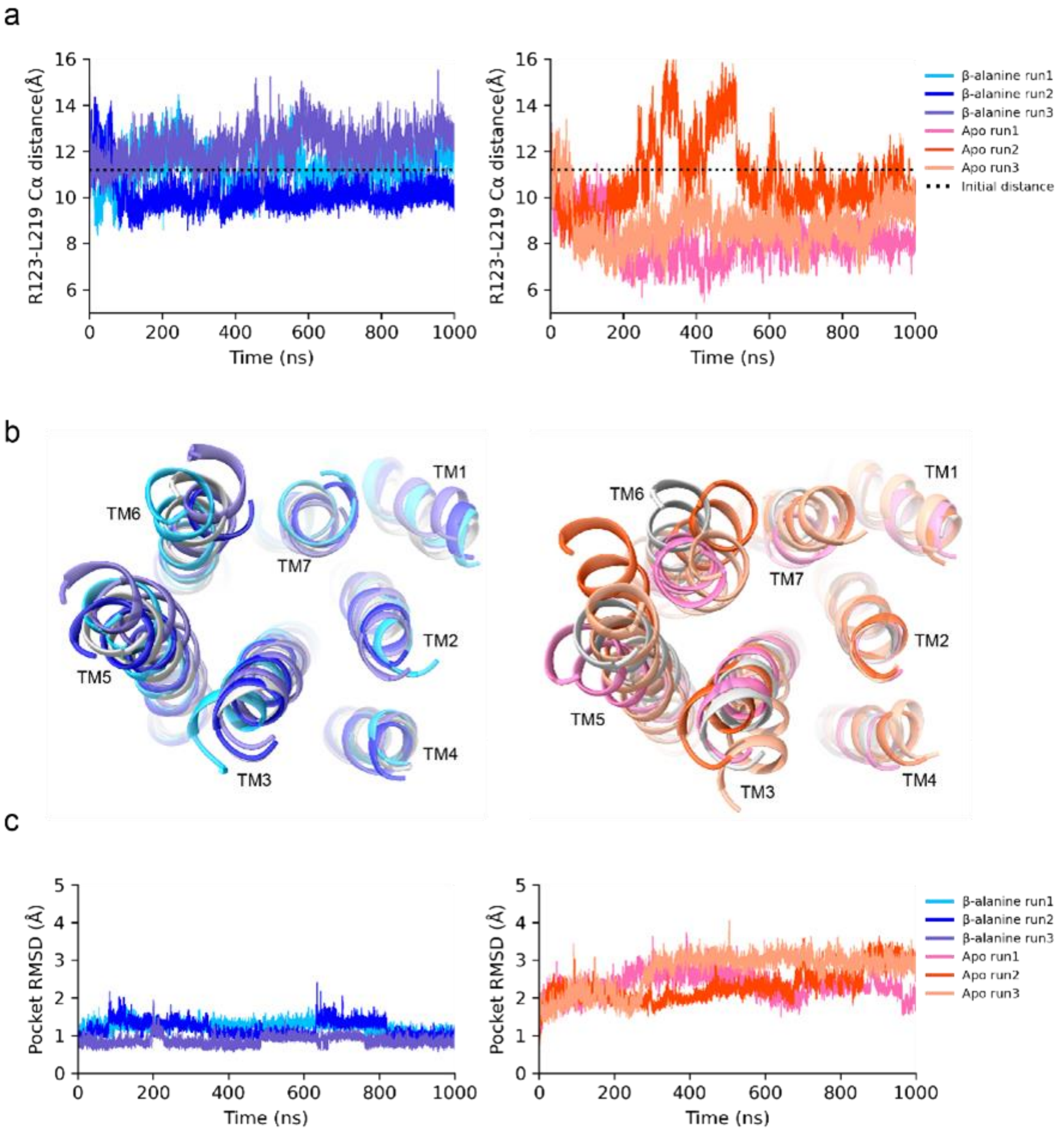

Supplementary Fig. 15 | MD simulations of MrgD in the apo and β-alanine-bound states

a TM6 displacement shown by the Cα distance between $R123^{3.50}$ and $L219^{6.33}$. The initial distances measured in the cryo-EM models were marked as dashed lines. The β-alanine-bound state is shown left. The apo state is shown right. **b** Comparison of the helix arrangement on the cytoplasmic side shown by superimposing the active state structure of MrgD (grey) on the last MD snapshots of the β-alanine-bound state (left) and the apo state (right). The coloring is the same as in **a**. **c** RMSD time course of the atom coordinates of the residues comprising the binding pocket (R103, C164, C175, D179, W241, Y245, and W246), except hydrogens. RMSDs from the three independent runs were shown for each state. The cryo-EM models were used as the references for RMSD calculations. The coloring is the same as in **a**

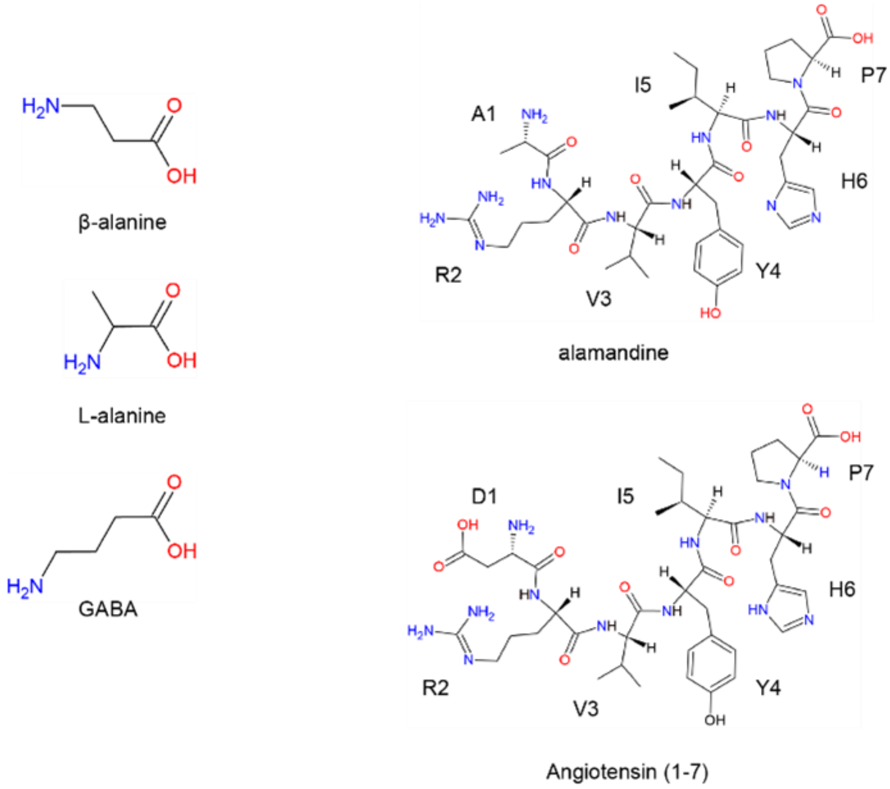

Supplementary Fig. 16 | Chemical structures of MrgD agonists

Two-dimensional representation of chemical structures of β-alanine, L-alanine, Gamma-Amino Butyric Acid (GABA), Alamandine and Angiotensin (1-7).

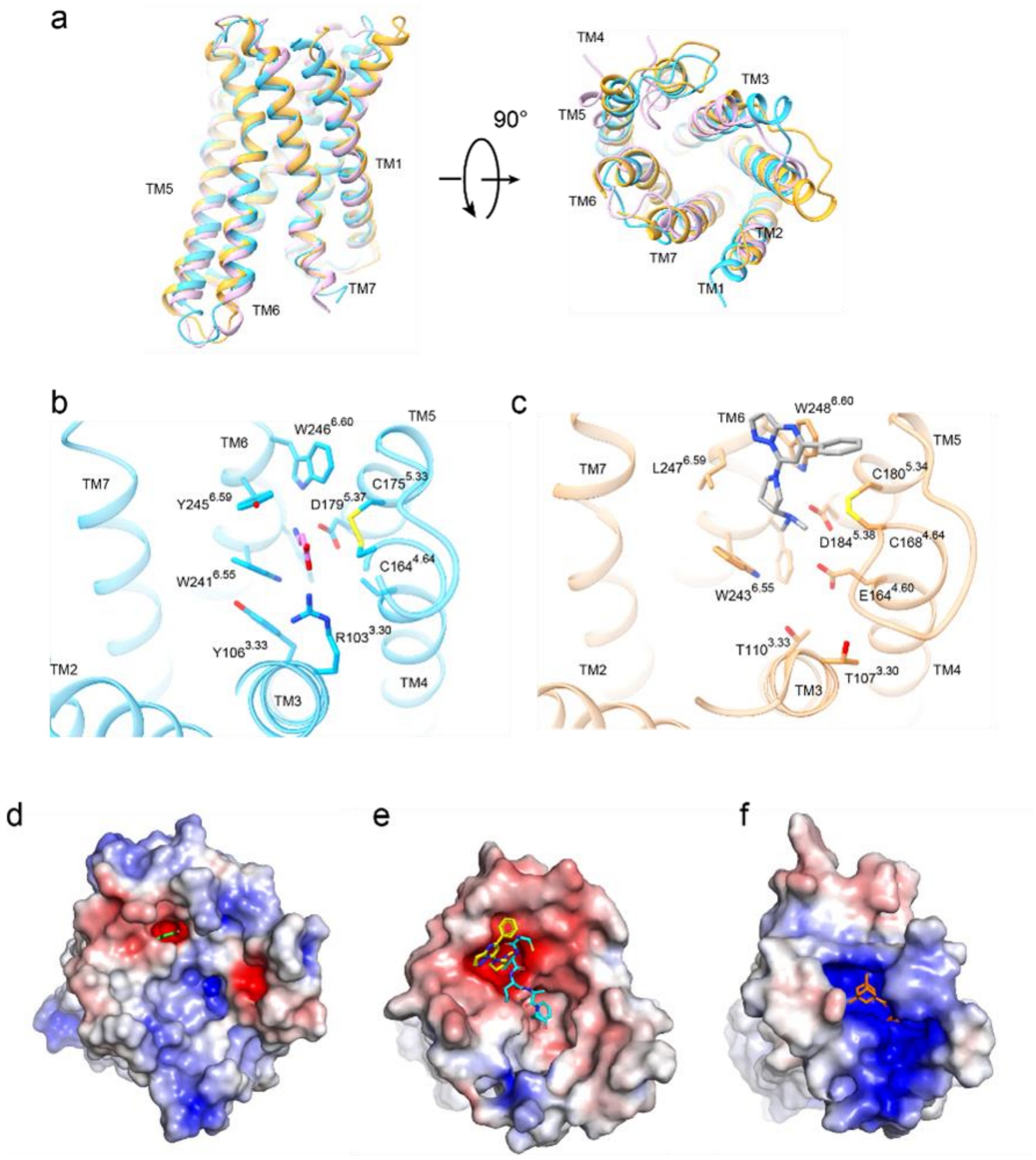

Supplementary Fig. 17 | Structural features of the MRGPR family

a Superposition of MrgD and MrgprX2 (orange, PDB code; 7S8O) and MrgprX4 (pink. PDB code; 7S8P). **b,c** Ligand-binding pocket. The side chains contributing to ligand binding are shown as stick models (b) MrgD, (c) MrgprX2. **d-f** Electrostatic surface representation of the (d) MrgD, (e) MrgprX2, (f) MrgprX4 extracellular pocket calculated using the APBS plugin in PyMOL, with β-alanine shown as a green stick and (R)-ZINC-3573 as a yellow stick, Cortistatin-14 as a cyan stick, and MS47134 as a pink stick.
