# Supplementary material for: Structural insight into the activation mechanism of MrgD with heterotrimeric Gi-protein revealed by cryo-EM
Source: Commun Biol. 2022 Jul 15;5:707. doi: 10.1038/s42003-022-03668-3 (PMC9287403; doi:10.1038/s42003-022-03668-3)
Supplement: Supplementary file 3 — Description of Additional Supplementary Files [file 42003_2022_3668_MOESM3_ESM.pdf]

Description of Additional Supplementary Files

File Name: Supplementary Data 1
Description: Source data underlying Figures 2f, 4c, and Supplementary Figures 1b and 11b.

File Name: Supplementary Data 2
Description: Source data underlying Figure 6d and Supplementary Figure 14
